# Iron deficiency without anemia: An underrecognized and undertreated clinical condition with special focus on colorectal cancer

**DOI:** 10.46989/001c.165952

**Published:** 2026-08-05

**Authors:** Naga P. Raja, Nagapavani Kandagari

**Affiliations:** 1 Oregon Health and Science University, Portland, Oregon, USA

**Keywords:** iron deficiency, non-anemic iron deficiency, ferritin, hepcidin, oral iron, intravenous iron, colorectal cancer, fatigue, iron deficiency with out anemia, IDNA

## Abstract

Iron deficiency is the most common micronutrient deficiency worldwide, affecting approximately 2 billion individuals. Iron deficiency without anemia (IDNA), the stage in which iron stores are depleted but hemoglobin remains within the reference range, is estimated to affect approximately 1-2 billion individuals, although the precise prevalence varies by population, ferritin threshold, and inflammatory status. IDNA remains underrecognized because diagnostic evaluation often focuses on hemoglobin concentrations rather than iron stores. A growing body of evidence demonstrates that IDNA is associated with clinically significant fatigue, impaired cognitive function, anxiety, reduced exercise capacity, and diminished quality of life. Randomized controlled trials and meta-analyses of iron repletion in iron-deficient non-anemic cohorts demonstrate clinically meaningful reductions in fatigue. Emerging observational data have also identified an association between iron deficiency and colorectal neoplasia in selected higher-risk populations such as older adults, men, and postmenopausal women. However, the magnitude of this risk and the role of routine endoscopic evaluation in patients with IDNA remain incompletely defined. This narrative review examines the epidemiology, pathophysiology, origins of under-recognition, clinical manifestations, diagnostic approach, treatment, association with colorectal cancer, and future directions for IDNA in adults.

## Introduction

Iron is an essential micronutrient involved in oxygen transport, mitochondrial energy production, DNA synthesis, and enzymatic reactions throughout the body.^1,2^ Iron deficiency exists on a continuum, progressing from depleted iron stores to iron-deficient erythropoiesis and, ultimately, iron deficiency anemia (IDA).^1,2^ The intermediate stage - iron deficiency without anemia (IDNA) - is far more common than IDA, yet it has historically received less clinical attention.^3,4^

Recent evidence has challenged the assumption that IDNA is a benign or subclinical condition. Randomized controlled trials and meta-analyses now demonstrate measurable symptomatic and functional consequences of IDNA, and expert consensus panels have issued recommendations supporting its treatment.^4,5^ Furthermore, emerging data on the association between IDNA and gastrointestinal malignancy raise important questions about the appropriate endoscopic workup of these patients.^6,7^ This review synthesizes the current state of knowledge regarding IDNA in adults.

## The Continuum of Iron Deficiency - From Depleted Stores to Anemia

Figure 1 illustrates the three-stage progression of iron deficiency from the iron-replete state through IDNA to IDA, with corresponding changes in key laboratory values (ferritin, transferrin saturation [TSAT], hemoglobin, mean corpuscular volume [MCV], soluble transferrin receptor [sTfR], reticulocyte hemoglobin content [CHr/Ret-He], and hepcidin), clinical manifestations at each stage, and approximate global prevalence estimates. The hemoglobin threshold separating IDNA from IDA is indicated (World Health Organization: 12 g/dL for women, 13 g/dL for men). Footnotes address the limitations of sTfR and the use of higher ferritin thresholds in inflammatory states.

**Figure:**
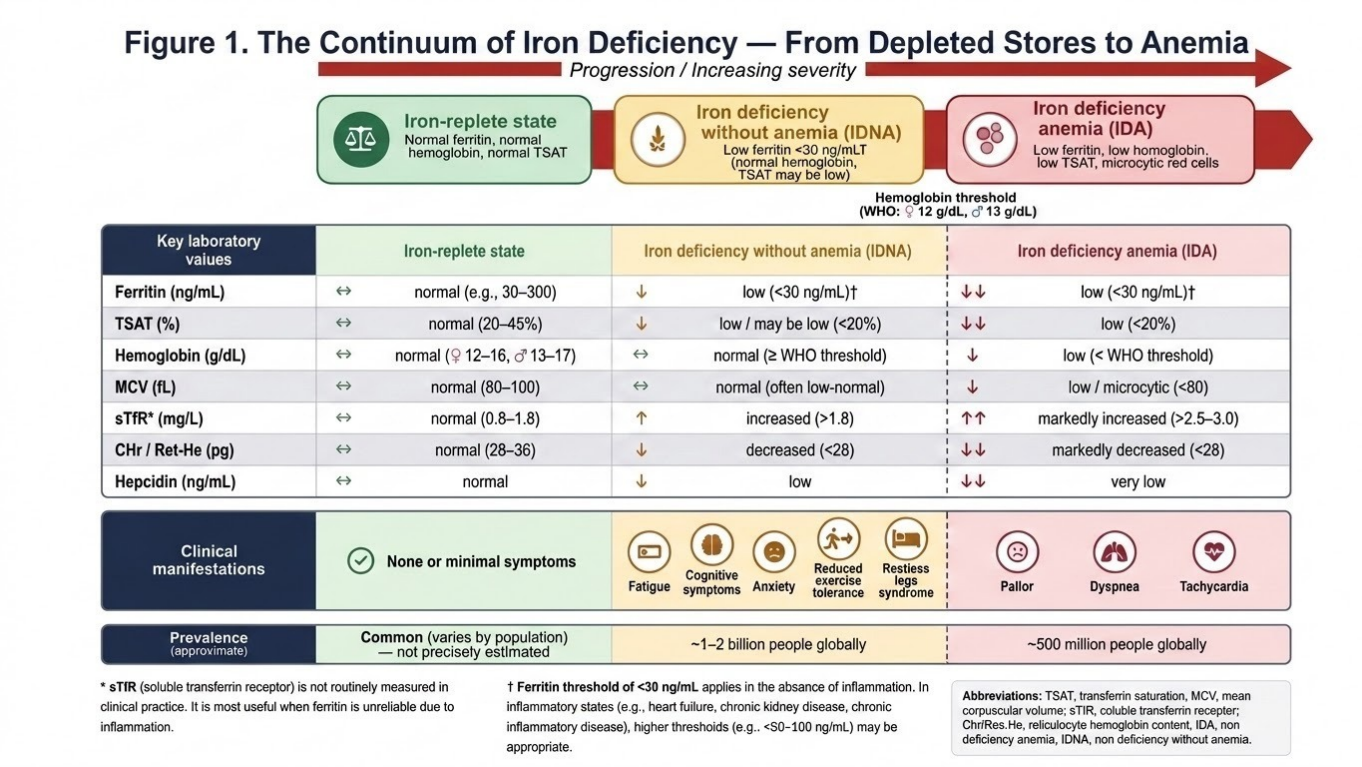


## Epidemiology

Iron deficiency is the most common nutritional deficiency globally, affecting approximately 2 billion people.^1,3^ IDNA represents a large subset of this global burden and is estimated to affect approximately 1-2 billion individuals, although precise estimates depend on population, ferritin cutoff, age, sex, pregnancy status, and the presence of inflammation.^1,3,8^

Absolute iron deficiency affects approximately 14% of adults in the United States.^1^ In high-income countries, IDNA disproportionately affects premenopausal women; approximately 38% of nonpregnant reproductive-age women have iron deficiency without anemia, compared with about 13% with iron-deficiency anemia.^1^ During the third trimester of pregnancy, iron deficiency affects up to 84% of pregnant women.^1^

Other at-risk groups include frequent blood donors, endurance athletes, individuals with chronic inflammatory conditions, and those with restrictive diets.^1,2^ Iron deficiency is especially prevalent among women and girls; applying current standards for ferritin reference ranges, approximately 40% of females aged 12-21 years meet criteria for iron deficiency.^3^ Additional risk factors include nonsteroidal anti-inflammatory drug use, inflammatory bowel disease (IBD), chronic kidney disease (CKD), heart failure, and cancer.^1^

The true prevalence of IDNA is likely underestimated because routine screening in nonpregnant adults is not universally recommended, laboratory thresholds vary, and many affected individuals are asymptomatic or attribute their symptoms to other causes.^1,4,8^

## Pathophysiology

Total body iron in adults is approximately 3-4 g, distributed among hemoglobin, storage forms (ferritin and hemosiderin), myoglobin, and iron-containing enzymes.^1,2^ Iron homeostasis is tightly regulated at the level of intestinal absorption and macrophage recycling, primarily through the hepcidin-ferroportin axis.^1,9^

IDNA can be categorized into two broad, and sometimes overlapping, mechanisms: absolute (quantitative) iron deficiency and functional or relative (qualitative) iron deficiency. In absolute iron deficiency, total body iron stores are depleted, ferritin is low, and hepcidin production is appropriately suppressed as a compensatory response to increase intestinal absorption and mobilize iron from macrophages.^1,9^ Common causes include menstrual blood loss, gastrointestinal blood loss, pregnancy, blood donation, reduced dietary intake, and malabsorption.

In functional or relative iron deficiency, total body iron stores may be normal or increased, but circulating iron availability is inadequate because iron is sequestered within the reticuloendothelial macrophage system. This pattern is driven largely by inflammatory upregulation of hepcidin, particularly through interleukin-6 signaling. Increased hepcidin degrades ferroportin in enterocytes and macrophages, reducing intestinal iron absorption and trapping iron in macrophages and the spleen. This is the same biological pathway that underlies anemia of chronic disease; before anemia develops, it may present clinically as IDNA with low TSAT and normal or elevated ferritin.^1,2,9^

Hepcidin also explains the absorption kinetics of oral iron. A single large oral iron dose increases plasma iron and stimulates hepcidin, which can suppress fractional iron absorption from subsequent doses for approximately 24-48 hours.^10^ This physiology supports lower-dose and alternate-day oral iron strategies for many patients with IDNA.

A distinct and rare cause of hepcidin-mediated iron restriction is iron-refractory iron deficiency anemia (IRIDA), most commonly due to TMPRSS6 variants causing inappropriately increased hepcidin synthesis. Although classically described as microcytic anemia, milder or earlier presentations may include iron deficiency before overt anemia. Recognition is important when iron deficiency is unexplained, lifelong, familial, or poorly responsive to oral iron.^11^

In IDNA, erythropoiesis is not yet compromised to the point of reducing hemoglobin below the normal range. However, non-erythropoietic iron-dependent processes - including mitochondrial oxidative phosphorylation, neurotransmitter synthesis (dopamine, serotonin), and skeletal muscle function - may already be impaired.^1^ This explains why fatigue, cognitive dysfunction, and exercise intolerance can manifest before frank anemia develops.

**Table 1.** Pathophysiologic Patterns of Iron Deficiency Relevant to IDNA

**Feature**	**Absolute (quantitative) iron deficiency**	**Functional/relative (qualitative) iron deficiency**
Iron stores	Depleted total body iron stores	Normal or increased stores but unavailable for erythropoiesis and tissues
Ferritin	Low, commonly <30 ng/mL in the absence of inflammation	Normal or elevated because ferritin is an acute-phase reactant
TSAT	Low or may become low as deficiency progresses	Typically low, often <20%
Hepcidin	Appropriately decreased	Inappropriately increased due to inflammation, especially IL-6
Typical settings	Menstrual or GI blood loss, pregnancy, donation, low intake, malabsorption	CKD, heart failure, IBD, infection, malignancy, chronic inflammatory disease
Clinical implication	Usually responds to oral iron if absorption and adherence are adequate	May require treating inflammation and/or IV iron; ferritin alone can be misleading

## Origins of Under-Recognition and Under-Treatment

Despite affecting an estimated 1-2 billion people globally, IDNA remains poorly recognized and undertreated.^1,3,8^ Several interrelated factors perpetuate this diagnostic and therapeutic gap.

### Attention Classically Triggered by Iron Deficiency Anemia

Historically, clinical recognition of iron deficiency has been triggered by manifestations of overt IDA, including pallor, exertional dyspnea, pica, koilonychia, and tachycardia. In contrast, patients with IDNA frequently present before these findings develop, and their symptoms may not prompt iron testing unless clinicians deliberately consider iron stores rather than hemoglobin alone.

### Non-Specific and Gradual Symptom Onset

Symptoms of IDNA - fatigue, reduced exercise tolerance, impaired concentration, irritability, and mood disturbance - are non-specific and often develop gradually. Patients and clinicians may attribute them to stress, aging, depression, hypothyroidism, sleep disorders, overtraining, or competing comorbidities, delaying diagnosis and treatment.^1^

### Hemoglobin-Centric Diagnostic Paradigm

The traditional clinical approach equates iron deficiency with anemia, meaning that iron studies are often not ordered unless hemoglobin is low. Multiple guidelines and professional groups recommend against using hemoglobin or hematocrit alone to evaluate susceptible patients, yet this practice persists.^4,9^

### Lack of Consensus on Ferritin Thresholds

Ferritin cutoffs for diagnosing iron deficiency vary widely across guidelines, from approximately 12-15 ng/mL to 45-50 ng/mL, creating diagnostic confusion in primary care.^8^ A study in JAMA Network Open demonstrated that the choice of ferritin threshold has profound implications: lower cutoffs miss a meaningful proportion of truly iron-deficient patients, whereas higher cutoffs may increase overdiagnosis.^8^

### Inadequate Laboratory Reference Ranges

Many clinical laboratories still report ferritin reference ranges with lower limits as low as 10–15 ng/mL, which are derived from population distributions rather than physiological thresholds for iron sufficiency. A recent pre-post intervention study demonstrated that optimizing the lower limit of normal for ferritin in the electronic health record (from gender-specific cutoffs of 10 ng/mL for women and 22 ng/mL for men to a universal cutoff of 30 ng/mL) significantly improved iron deficiency diagnosis and treatment patterns.^12^

### Ferritin as an Acute-Phase Reactant

Ferritin may be falsely elevated in infection, inflammation, liver disease, malignancy, CKD, heart failure, and IBD.^1,9^ In such settings, TSAT, sTfR, CHr/Ret-He, or the transferrin receptor-ferritin index may help identify functional iron deficiency when ferritin alone is misleading.

### Normalization of Symptoms in High-Risk Populations

Women and girls are disproportionately affected, with 40% of females aged 12–21 years meeting criteria for iron deficiency.^3^ Symptoms such as fatigue and reduced exercise tolerance are frequently normalized in menstruating women, athletes, and adolescents, delaying diagnosis and treatment.

## Clinical Manifestations

### Fatigue

Fatigue is the most consistently reported symptom of IDNA and the outcome with the strongest evidence base. A systematic review and meta-analysis of 18 randomized controlled trials (n=1,170) found that oral iron supplementation significantly reduced self-reported fatigue in non-anemic iron-deficient adults (standardized mean difference [SMD]-0.38; 95% CI, -0.52 to -0.23).^5^ A Cochrane-derived meta-analysis of intravenous iron in IDNA also demonstrated significant fatigue reduction (SMD -0.30; 95% CI, -0.52 to -0.09).^13^ An earlier meta-analysis of 6 RCTs similarly confirmed improvement in fatigue with iron replacement in IDNA (pooled effect size 0.33; 95% CI, 0.17-0.48).^14^

### Physical Capacity and Exercise Performance

Evidence regarding objective measures of physical performance is less consistent than for fatigue. Oral iron supplementation was not associated with significant improvement in maximal oxygen consumption in the Houston et al meta-analysis (SMD 0.11; 95% CI, -0.15 to 0.37).^5^ However, intravenous iron demonstrated a significant improvement in peak oxygen consumption in the Cochrane review, although heterogeneity limits confidence.^13^

This difference could be explained by the fact that intravenous iron achieves more rapid and complete iron repletion than oral formulations, resulting in substantially larger increases in ferritin and iron availability. Consequently, IV iron may be more likely to restore iron-dependent skeletal muscle and mitochondrial function sufficiently to influence exercise performance, whereas oral iron often produces slower and less complete correction of iron deficiency.^13^

### Cognitive and Psychiatric Outcomes

A 2025 meta-analysis of 18 studies (n=1,340) in non-anemic children, adolescents, and menstruating women found that iron supplementation improved anxiety, cognitive intelligence, short-term memory, and physical well-being in RCTs.^15^ Notably, these effects were observed only in participants with confirmed iron deficiency, suggesting specificity of the intervention. Pre-post studies showed even larger improvements in depression (d=0.93), fatigue (d=1.01), and overall psychiatric symptoms (d=1.13).^15^

A recent cross-sectional study of 209 adolescents found that IDNA was associated with lower striatal iron content and altered basal ganglia structure and function, which correlated with increased psychiatric symptom severity and worse neuropsychiatric performance.^16^

### Other Manifestations

Additional symptoms associated with IDNA include restless legs syndrome, pica, irritability, depression, difficulty concentrating, lightheadedness, and exercise intolerance.^1^ Prevalence and severity vary by age, comorbidities, and the rate at which iron deficiency develops.

## Diagnosis

### Ferritin as the Primary Biomarker

Serum ferritin remains the most widely recommended initial test for iron deficiency.^1,9^ A ferritin level below 30 ng/mL is commonly endorsed for diagnosing iron deficiency in the absence of inflammation, offering excellent specificity.^1,12^ The American Gastroenterological Association recommends a threshold of 45 ng/mL, which balances high sensitivity of 85% while maintaining high specificity of 92%.^17^ A Delphi consensus panel achieved agreement that a ferritin level below 30 ng/mL should be considered diagnostic of iron deficiency in all adult patients, regardless of gender or comorbid status.^12^

### Limitations of Ferritin

Ferritin is an acute-phase reactant and may be falsely elevated in infection, inflammation, liver disease, or malignancy.^1,9^ In these contexts, additional markers such as transferrin saturation (TSAT) below 20%, soluble transferrin receptor (sTfR), reticulocyte hemoglobin content (CHr/Ret-He), or the transferrin receptor-ferritin index may be helpful.^1,9^ In patients with chronic inflammation, a ferritin threshold of 100 ng/mL or higher may be appropriate depending on the clinical context.^1,9^

### Defining “Without Anemia”

IDNA is defined as iron deficiency, usually evidenced by ferritin below 15-30 µg/L or an otherwise convincing iron-restricted pattern, in the presence of a normal hemoglobin concentration (>12.0 g/dL in women and >13.0 g/dL in men).^1,9^

### Evaluation for Underlying Cause

An underlying etiology for iron deficiency should always be sought. In premenopausal women, menstrual blood loss is the most common cause. In men and postmenopausal women, gastrointestinal evaluation — including upper and lower endoscopy — is recommended to exclude occult blood loss, celiac disease, or malignancy.^17^ The AGA recommends bidirectional endoscopy for iron deficiency anemia; the threshold for endoscopic evaluation in IDNA is less clearly defined but should be guided by clinical suspicion (see section on Colorectal Cancer Risk below).^17,18^

## Treatment

When an underlying cause of IDNA is identified, it should be addressed according to the relevant disease-specific evaluation and management guidelines. Gastrointestinal blood loss, heavy menstrual bleeding, malabsorption, inflammatory disorders, dietary insufficiency, medication-related bleeding, and malignancy require etiology-specific evaluation and management. The discussion below focuses on the symptomatic approach of iron repletion after appropriate etiologic evaluation.

### Oral Iron

Oral iron remains the first-line treatment for most patients with IDNA.^1,4^ The 2025 Lancet Haematology expert consensus panel issued a strong recommendation for daily oral iron in nonpregnant adults with iron deficiency without anemia to improve fatigue, ferritin, and hemoglobin concentrations.^4^ A 2025 JAMA review similarly supports oral iron as first-line therapy.^1^

Alternate-day dosing has emerged as a preferred strategy based on evidence that oral iron increases hepcidin and can reduce fractional iron absorption from subsequent doses.^10,19^ Morning dosing with 60-120 mg elemental iron on alternate days may optimize absorption and tolerability for many patients.^19^ Daily supplementation may still be reasonable when symptoms are severe, adherence is easier with daily dosing, or gastrointestinal side effects are absent or tolerable.^20^

Gastrointestinal adverse effects, including nausea, constipation, and abdominal pain, are the main barrier to adherence. Strategies to improve tolerability include alternate-day dosing, lower-dose formulations, taking iron with a small amount of food when needed, and avoiding tea, coffee, calcium-containing foods, and calcium supplements near the time of iron administration.^1,9^

### Intravenous Iron

Intravenous iron is appropriate for patients who cannot tolerate oral iron, have malabsorptive conditions such as celiac disease, inflammatory bowel disease, or post-bariatric surgery, have ongoing losses exceeding the capacity of oral replacement, or require rapid correction.^1,2^ Modern IV iron formulations have favorable safety profiles, with serious adverse events, including anaphylaxis, occurring rarely.^21^

A notable adverse effect of ferric carboxymaltose is hypophosphatemia, which can be clinically significant and prolonged. Ferric derisomaltose is associated with a lower incidence of this complication compared with ferric carboxymaltose.^21,22^

The Cochrane review of IV iron for non-anemic iron-deficient adults found low-quality evidence for a small hemoglobin increase, significant fatigue reduction, and improvement in peak oxygen consumption, with uncertain effects on quality of life.^13,23^

### Comparison of Iron Formulations

Table 2 summarizes commonly used oral and intravenous iron formulations, including dosing, advantages, disadvantages, and the strength of evidence in IDNA.^1,4,13,19–23^

**Table 2.** Comparison of Commonly Used Oral and Intravenous Iron Formulations Relevant to IDNA.

**Formulation**	**Route**	**Elemental Iron per Dose**	**Dosing Frequency**	**Key Advantages**	**Key Disadvantages**	**Evidence in IDNA**
Ferrous sulfate	Oral	65 mg per tablet	Daily or alternate-day	Low cost (~USD 4–10/course), widely available, gold standard	GI side effects (up to 70%); metallic taste, constipation	Strong (multiple RCTs)
Ferrous gluconate	Oral	35–38 mg per tablet	Daily or alternate-day	May be better tolerated than sulfate	Lower elemental iron per dose; limited head-to-head data	Limited
Ferric maltol (Accrufer)	Oral	30 mg per capsule	Twice daily	FDA-approved for iron deficiency (with or without anemia); better GI tolerability	Higher cost; requires empty stomach; minimum 12 weeks	Emerging (FDA-approved for ID broadly)
Ferric carboxymaltose (Injectafer)	IV	Up to 750–1000 mg/infusion	2 infusions, ≥7 days apart (total 1500 mg)	Rapid repletion; well-studied	Hypophosphatemia risk (47–75%); severe hypophosphatemia in ~11%; cost (~$3,681/course)	Moderate (Cochrane review data)
Ferric derisomaltose (Monoferric)	IV	Up to 1000 mg single dose	Single infusion	Full replacement in one visit; significantly lower hypophosphatemia risk (~4–8%) versus FCM	Cost (~$4,214/course); IV access required	Moderate
Ferumoxytol (Feraheme)	IV	510 mg per infusion	2 infusions 3–8 days apart (total 1020 mg)	Rapid infusion (15–30 min); can give full dose in 1–2 visits	Boxed warning (serious hypersensitivity); hypotension (1.9%); MRI interference for ≤3 months	Limited in IDNA
Low-molecular-weight iron dextran (INFeD)	IV	Up to 1000 mg total dose infusion	Single total dose infusion (after 25 mg test dose)	Lowest cost IV option (~$405/course); full repletion in one session	Boxed warning (anaphylaxis); requires test dose; delayed reactions	Limited in IDNA
Iron sucrose (Venofer)	IV	100–300 mg per infusion	5 infusions over 14 days or multiple sessions	No test dose required; no boxed warning; well-established safety in CKD	Multiple infusions needed; FDA-approved only for CKD	Limited (2 Cochrane-included RCTs for IDNA)

### Treatment Targets and Monitoring

There is no universally agreed-upon ferritin target for treatment completion. Expert consensus generally recommends treating until ferritin exceeds 50-100 ng/mL and TSAT normalizes above 20%.^1,4^ Ferritin can be rechecked 8-12 weeks after initiating oral iron or 4-8 weeks after IV iron infusion.^1^

### Special Populations

Heart failure: Iron deficiency, with or without anemia, is highly prevalent in heart failure and is independently associated with worse functional capacity and outcomes. IV iron has been shown to improve symptoms and reduce heart failure hospitalizations in selected populations and is recommended by major heart failure guidelines.^1,4^

Pregnancy: Iron deficiency in pregnancy is associated with adverse maternal and neonatal outcomes. Treatment thresholds and strategies differ from the nonpregnant population and are addressed in dedicated guidelines.^4^

Athletes: Endurance athletes are at increased risk of IDNA due to exercise-induced hepcidin changes, hemolysis, and gastrointestinal losses. Iron supplementation may improve performance metrics in iron-deficient athletes, although evidence is mixed.^1^

Chronic kidney disease: Iron deficiency is prevalent in CKD and may be masked by elevated ferritin levels due to chronic inflammation. In this setting, TSAT and disease-specific thresholds are often required to guide therapy.^1,2^

## Iron Deficiency Without Anemia and Colorectal Cancer Risk

Recent epidemiologic evidence indicates a clinically relevant association between IDNA and gastrointestinal pathology, with the highest malignancy risk observed in selected high-risk cohorts such as older adults, men, and postmenopausal women.^6,7,18^

### Potential Biological Links Between Iron Deficiency and Colorectal Cancer

The relationship between iron deficiency and colorectal cancer is biologically complex. Iron can promote inflammation, oxidative stress, and tumor growth in some contexts, yet systemic iron deficiency may also impair antitumor immunity and worsen host resilience.^24,25^

Impaired immunosurveillance is one proposed mechanism. Iron is required for natural killer cell cytotoxicity, T-cell proliferation, and macrophage function; deficiency may compromise antitumor immune responses.^26,27^ Iron deficiency may also contribute to oxidative stress and genomic instability through impaired antioxidant enzyme function and DNA repair.^28^

Colorectal cancer cells can remodel iron metabolism by increasing iron-import proteins such as transferrin receptor 1 and divalent metal transporter 1 and reducing ferroportin, thereby retaining intracellular iron to support proliferation even when the host is systemically iron deficient.^25,29,30^ Recent work has also identified a heme-succinate dehydrogenase-coenzyme Q axis that allows colorectal cancer cells to buffer iron-induced oxidative stress.^31^

Clinical data support the relevance of this biology. In a 2025 single-center cohort study of 1,003 colorectal cancer patients, iron deficiency was present in approximately half and was associated with larger tumor diameter, more advanced stage, poorer differentiation, increased lymphovascular invasion, and inferior response to neoadjuvant therapy.^28^

### Prevalence of Gastrointestinal Malignancy in IDNA

The prevalence of gastrointestinal malignancy among patients with IDNA appears to be low in unselected populations, but available evidence suggests that risk is not uniform and may be clinically meaningful in selected higher-risk subgroups. A 2020 systematic review and meta-analysis, including five studies and 3,329 participants, reported an overall pooled gastrointestinal malignancy prevalence of 0.38% among patients with IDNA. However, subgroup estimates were higher among older patients, men and postmenopausal women, and non-screening populations, while malignancy prevalence was very low among premenopausal women.^6^

Subsequent cohort studies have provided additional, although variable, estimates of risk. In a 2023 Western Australian multicenter cohort of 584 patients undergoing endoscopic evaluation, the malignancy rate was significantly higher in patients with iron deficiency anemia than in those with IDNA (8.76% versus 1.20%, P<0.01). Anemia and male gender were significant predictors of malignancy. Importantly, more than 60% of patients, including those without anemia, had identifiable gastrointestinal pathology on endoscopy, suggesting that IDNA may still be associated with clinically relevant gastrointestinal disease even when malignancy is uncommon.^32^ In contrast, a 2026 Jordanian retrospective study of 480 patients found no significant difference in gastrointestinal malignancy rates between IDA and IDNA (1.8% versus 2.4%). Colorectal adenocarcinoma was the most common malignancy, and increasing age was the only significant predictor.^7^

Data on young-onset colorectal cancer are stronger for iron deficiency anemia than for IDNA specifically. In adults aged 18–49 years, iron deficiency anemia was associated with a higher 5-year cumulative incidence of colorectal cancer compared with individuals without iron deficiency anemia, with risk differences increasing with age and being substantially higher in men.^33^ Although these findings cannot be directly extrapolated to all patients with IDNA, they reinforce the broader clinical principle that iron deficiency may serve as a marker of occult gastrointestinal pathology, particularly in men and older adults.

Taken together, current evidence does not support universal bidirectional endoscopy for all patients with IDNA. However, IDNA should not be dismissed as benign when it is persistent, recurrent, unexplained, or present in higher-risk individuals. A risk-adapted approach to gastrointestinal and colorectal cancer evaluation is appropriate, incorporating age, gender, menopausal status, gastrointestinal symptoms, family history, degree and persistence of iron deficiency, and response to iron repletion.

### Guideline Gaps and Clinical Implications

Current guidelines provide clear recommendations for the gastrointestinal evaluation of iron deficiency anemia but offer limited guidance for non-anemic iron deficiency. The AGA strongly recommends bidirectional endoscopy for men and postmenopausal women with IDA, while available observational data and endoscopic literature highlight the ongoing uncertainty regarding when IDNA alone should prompt bidirectional endoscopy.^17,18^ As a result, there remains no standardized algorithm defining when IDNA alone should prompt bidirectional endoscopy.

Given the low overall prevalence of gastrointestinal malignancy in unselected IDNA populations, available data do not support routine bidirectional endoscopy for all patients with IDNA. Instead, endoscopic evaluation should be individualized, with greater consideration in older adults, men, postmenopausal women, patients with persistent or recurrent iron deficiency, positive fecal testing, gastrointestinal symptoms, family history, or other colorectal cancer risk factors.^7,17,18^

Fecal immunochemical testing (FIT) may help prioritize colonoscopy in iron-deficient patients. A systematic review and meta-analysis found that FIT detected colorectal cancer and advanced precancerous neoplasia in iron-deficient patients with sensitivities of 90.7% and 49.3% and specificities of 81.0% and 82.4%, respectively.^34^ However, only one included study specifically enrolled a non-anemic iron deficiency cohort; therefore, prospective validation is needed before FIT can be routinely recommended as a triage strategy for IDNA.^34^

## Future Directions

## Several important questions remain unanswered

1. Optimal ferritin thresholds for diagnosis and treatment initiation in asymptomatic individuals remain debated, with recommended cutoffs varying across guidelines.^8^

2. Long-term outcomes of treating IDNA, including effects on cardiovascular events, pregnancy outcomes, neurodevelopment, cognitive function, and quality of life, require further study.

3. Endoscopic evaluation criteria for IDNA should be defined by prospective studies, particularly in older patients, men, and postmenopausal women, where malignancy risk appears higher.^6,7^

4. Novel oral iron formulations and strategies to improve tolerability and adherence warrant additional randomized trials.

5. Point-of-care ferritin testing could facilitate broader screening and earlier identification of IDNA in primary care and community settings.

6. The iron-cancer axis, including whether iron deficiency itself promotes colorectal cancer progression or impairs treatment response, requires further clinical investigation.^26,28^

7. FIT as a triage strategy for endoscopic evaluation in IDNA requires prospective validation specifically in non-anemic iron-deficient individuals.^34^

## Conclusion

IDNA is a highly prevalent condition with clinically meaningful consequences, particularly fatigue, impaired physical capacity, cognitive symptoms, and anxiety. Recent evidence and expert consensus recommendations support active identification and treatment of IDNA. Oral iron, often dosed on alternate days, remains first-line therapy, with intravenous iron reserved for intolerance, malabsorption, ongoing losses, or need for rapid repletion. The emerging association between IDNA and gastrointestinal malignancy, particularly among older adults, men, and postmenopausal women, highlights the need for clearer endoscopic evaluation criteria. Clinicians should maintain a low threshold for ferritin testing in at-risk populations and should investigate the underlying cause of iron deficiency rather than treating the laboratory abnormality in isolation.

### Disclosures

Both authors disclose no financial disclosures.

### Authors’ Contributions (CRediT Taxonomy)

Conceptualization: Naga P. Raja (Lead). Methodology: Naga P. Raja (Lead). Investigation: Naga P. Raja (Equal), Nagapavani Kandagari (Equal). Data curation: Naga P. Raja (Equal), Nagapavani Kandagari (Equal). Visualization: Naga P. Raja (Equal), Nagapavani Kandagari (Equal). Software: Naga P. Raja (Lead). Writing – original draft: Naga P. Raja (Lead). Writing – review & editing: Naga P. Raja (Equal), Nagapavani Kandagari (Equal). Supervision: Naga P. Raja (Lead).

### Competing Interests

The authors declare that they have no competing interests.

### Ethical Conduct Approval

Not applicable. This manuscript is a narrative review of the published literature and did not involve human participants, animals, identifiable patient data, or original experimental research.

## Data Availability

The data supporting the findings of this review are derived from previously published studies. Additional information and supporting materials are available from the corresponding author upon reasonable request.
